# Safety and Immunogenicity of a 4-Component Toxoid-Based *Staphylococcus aureus* Vaccine in Rhesus Macaques

**DOI:** 10.3389/fimmu.2021.621754

**Published:** 2021-02-25

**Authors:** Arundhathi Venkatasubramaniam, Grant Liao, Eunice Cho, Rajan P. Adhikari, Tom Kort, Frederick W. Holtsberg, Karen E. Elsass, Dean J. Kobs, Thomas L. Rudge, Keith D. Kauffman, Nickiana E. Lora, Daniel L. Barber, M. Javad Aman, Hatice Karauzum

**Affiliations:** ^1^ Integrated BioTherapeutics, Rockville, MD, United States; ^2^ Batelle - West Jefferson, West Jefferson, OH, United States; ^3^ Laboratory of Parasitic Diseases, T Lymphocyte Biology Section, National Institute of Allergy and Infectious Diseases, National Institutes of Health, Bethesda, MD, United States

**Keywords:** safety, immunogenicity, multi-component, toxoid, staphylococcal, neutralizing antibodies, CD4 T cells response

## Abstract

*Staphylococcus aureus* is a leading cause of significant morbidity and mortality and an enormous economic burden to public health worldwide. Infections caused by methicillin-resistant *S. aureus* (MRSA) pose a major threat as MRSA strains are becoming increasingly prevalent and multi-drug resistant. To this date, vaccines targeting surface-bound antigens demonstrated promising results in preclinical testing but have failed in clinical trials. *S. aureus* pathogenesis is in large part driven by immune destructive and immune modulating toxins and thus represent promising vaccine targets. Hence, the objective of this study was to evaluate the safety and immunogenicity of a staphylococcal 4-component vaccine targeting secreted bi-component pore-forming toxins (BCPFTs) and superantigens (SAgs) in non-human primates (NHPs). The 4-component vaccine proved to be safe, even when repeated vaccinations were given at a dose that is 5 to 10- fold higher than the proposed human dose. Vaccinated rhesus macaques did not exhibit clinical signs, weight loss, or changes in hematology or serum chemistry parameters related to the administration of the vaccine. No acute, vaccine-related elevation of serum cytokine levels was observed after vaccine administration, confirming the toxoid components lacked superantigenicity. Immunized animals demonstrated high level of toxin-specific total and neutralizing antibodies toward target antigens of the 4-component vaccine as well as cross-neutralizing activity toward staphylococcal BCPFTs and SAgs that are not direct targets of the vaccine. Cross-neutralization was also observed toward the heterologous streptococcal pyogenic exotoxin B. *Ex vivo* stimulation of PBMCs with individual vaccine components demonstrated an overall increase in several T cell cytokines measured in supernatants. Immunophenotyping of CD4 T cells *ex vivo* showed an increase in Ag-specific polyfunctional CD4 T cells in response to antigen stimulation. Taken together, we demonstrate that the 4-component vaccine is well-tolerated and immunogenic in NHPs generating both humoral and cellular immune responses. Targeting secreted toxin antigens could be the next-generation vaccine approach for staphylococcal vaccines if also proven to provide efficacy in humans.

## Introduction

*Staphylococcus aureus* (SA) is a gram-positive bacterial pathogen that is a leading cause of hospital and community-associated infections worldwide (1, 2). Currently, there is no vaccine or therapeutic developed against SA infections, and treatment is limited to antibiotics, which are not always successful due to the rise of antibiotic resistant strains of SA (3). Multiple vaccines and therapeutic candidates have been assessed for prevention of *S. aureus* infections. To date, all have targeted cell surface antigens, facilitated opsonophagocytosis and have functioned well in murine models, but either lacked efficacy or resulted in increased mortality in human clinical trials (4, 5). Recent studies have indicated that vaccination with a lethally irradiated USA300 whole cell preparation or with cell surface antigens enhances SA disease while vaccination with toxoids provided protection (6, 7). A different and novel approach hence would be to generate neutralizing antibodies to toxins secreted by SA, thus providing clinical protection versus sterile immunity. Toxins are among the key virulence factors that define SA, and typically act by damaging biological membranes leading to cell death or by interfering with receptor functions (8). SA toxins may be classified into three major groups - the pore-forming toxins (PFTs) (α-hemolysin, β-hemolysin, leukotoxins and phenol-soluble modulins (PSMs), exfoliative toxins (ETs) and family of superantigens (SAgs) (9). We have developed a four-component vaccine that consists of toxoids previously described in literature and that targets alpha-hemolysin, leukocidins, and superantigen toxins produced by *S. aureus*. Briefly, mutant forms of both Panton Valentine Leukocidin (PVL) subunits, LukS_mut9_ (LukS_T28F/K97A/S209A_) and LukF_mut1_ (LukF_K102A_), were identified as potential vaccine candidates and shown to confer protection in a mouse bacteremia model (10). We also designed an attenuated full-length Hla molecule Hla_H35L/H48L_, containing mutations in two histidine residues, H35 and H48 known to be critical for Hla oligomerization and showed it to be immunogenic as well as efficacious in a rabbit pneumonia model when immunized in combination with LukS_mut9_ and LukF_mut1_ (11). Further, in order to develop a vaccine component against superantigens, we engineered a single fusion protein TBA_225_ consisting of toxoid versions of TSST-1, SEB, and SEA and demonstrated its immunogenicity and protective efficacy in a mouse model of toxic shock (12). A fifth component LukAB_mut50_ that completes IBT-V02 was still under development during the time of the NHP safety study and hence not included.

In comparison to mice which are the typical animal model used to study SA vaccines due to their easy availability, non-human primates are more susceptible than mice to a majority of human-adapted pathogens. They are closest to humans in terms of anatomy and immune responses and highly sensitive to staphylococcal toxins such as superantigens and Hla and hence remain an important disease model for translating the discovery of treatments and vaccines into potential clinical outcomes (13–16). Hence, in the present study, we chose NHPs as the model of choice for our vaccine safety and immunogenicity study described in this article. We demonstrate that a 4-component vaccine is safe and immunogenic in NHPs, and that it elicits neutralizing and cross-neutralizing antibodies to several SA toxins as well as an antigen-specific CD4 T cell response of a Th1/Th17 phenotype.

## Materials and Methods

### Vaccine

The production and characterization of the individual components of the 4-component vaccine has been reported previously (10–12).

### Animals and Immunizations

Four male and four female Rhesus Macaques of Chinese origin were purchased from Covance Research Products Inc. (Alice, TX) and were housed in the testing facilities of Battelle, West Jefferson, OH. Animals ranged from 41–44 months of age with body weights ranging from 3.9 to 4.7 kg in weight for males and 4.2 to 4.5 kg for females on Day 0.

Prior to each dose administration, study animals were sedated by administering an intramuscular (IM) injection of ketamine (10 mg/kg). The fur was clipped from around all injection areas and the injection area was wiped with isopropyl alcohol, prior to dose administration. An IM injection of Alhydrogel (Al(OH)_3_) alone or 4-component vaccine of 400 μg (100 μg/component) formulated in 1,600 μg Al(OH)_3_ was administered in the thigh musculature on Days 0, and followed by three booster immunizations on days 21, 42, and 182. The injection site was marked with indelible ink for observation of the dose administration sites (Days 0, 21, and 42 only).

### Blood Specimen Collection

Blood specimens were collected on days -21, 0 (prime), 1, 21 (1^st^ booster), 42 (2^nd^ booster), and 63 and used for clinical pathology (hematology and serum chemistry parameters), and serology endpoints. Blood specimens collected at different timepoints related to study start were used to isolate PBMCs for flow cytometry.

### Hematology

The following hematology parameters were evaluated using the Advia 120 Hematology Analyzer: Absolute reticulocyte count, cell morphology, erythrocyte count (RBC), hematocrit (HCT), hemoglobulin (HGB), mean corpuscular hemoglobulin (MCH), mean corpuscular hemoglobulin concentration (MCHC), mean corpuscular volume (MCV), mean platelet volume (MPV), total leukocyte count (WBC), differential leukocyte count: neutrophils (NEUT), lymphocytes (LYMPH), monocytes (MONO), basophils (BASO), eosinophils (EOS), and large unstained cells (LUC).

### Serum Chemistry

Following serum chemistry parameters were evaluated using a Roche COBAS c501 Chemistry Analyzer: Alanine aminotransferase (ALT), Albumin, Albumin/Globulin (A/G) ratio, Alkaline phosphatase (ALP), Aspartate aminotransferase (AST), Bilirubin (direct), Bilirubin (total), Blood urea nitrogen (BUN), C-reactive protein, Calcium, Cholesterol, Chloride, Creatine kinase (CK), Creatinine, Gamma glutamyltransferase (GGT), Globulin, Glucose, Lactate dehydrogenase, Phosphorus, Potassium, Sodium, Protein (total), and Triglycerides (TRIG).

### Serum Cytokines

Serum cytokines were detected using Meso Scale Diagnostics (MSD) NHP biomarker multiplex assay kits. Samples or prepared calibrator standards provided in the kit were diluted along with reagent diluent in a provided MSD 96-well 10-Spot plate and incubated at room temperature with shaking for 1 h. The plates were then washed thrice with 1X Wash buffer followed by incubation with detection antibody solution at room temperature with shaking for 1 h. The plates were washed again with 1X Wash buffer followed by addition of 2X Read buffer T to each well. Then the plates were analyzed on an MSD instrument. The instrument measures the intensity of emitted light (which is proportional to the amount of analyte present in the sample) and provides a quantitative measure of each analyte in the sample. Data was analyzed with GraphPad PRISM v8.3.1.

### PBMC Isolation and Flow Cytometry

Blood for flow cytometry was obtained from NHPs at different timepoints related to study start. Aliquots were collected for serum separation prior to isolation of peripheral blood mononuclear cells (PBMCs). PBMC specimens were washed, counted using Guava PCA, and cryopreserved in CryoStor at a concentration of 1 × 10^7^ viable cells/ml. The resulting cell suspension was then divided equally into aliquots ≥1 ml and ≤2 ml and placed into a chilled cryofreezing container. Cryopreserved cells were thawed, washed and rested in X-Vivo 15 media + 10% FCS for 16 h. Cells were then incubated for 6 h at 37°C and 5% CO_2_ in X-Vivo 15 media supplemented with 10% FCS and Brefeldin A, and Monensin along with 400 µg/ml of 4-component vaccine (100 µg/ml of each Hla_H35L/H48L_, LukS_mut9_, LukF_mut1_, and TBA_225_), 1µg/ml SEB, or left unstimulated. Cells were stained with following fluorochrome-labelled antibodies: IFNγ (B27), CD4 (SK3), CD28 (CD28.2), CD8 (SK1), GM-CSF (BVD2-21C11), CD40L (24-31), IL-2 (MQ1-17H12), CD95 (DX2), CD3 (SP34-2), TNF (MAB11), CD153 (116614), IL-17A (ebio64DEC17), IL-21 (3A3-N2) and fixable dye eFluor 780 purchased from BD Biosciences (San Jose, CA), Biolegend (San Diego, CA), R&D Systems (Minneapolis, MN), Thermo Fisher/ebio. All samples were acquired on a BD Symphony A5 flow cytometer and analyzed using FlowJo software (Version 10, Tree Star). Statistical analysis was performed using non-parameteric t-test. GraphPad Prism 8.4.3.

### *Ex Vivo* Cytokine Release

Isolated PBMCs were stimulated either with 100 µg/ml of each individual vaccine component or were left unstimulated at 37°C and 5% CO_2_ in X-Vivo 15 media supplemented with 10% FCS. Supernatants were collected 24 h post stimulation and cytokines were assessed on a Luminex 200 using the NHP Th cytokine 14-plex ProcartaPlex Panel (ThermoFisher) according to the manufacturer’s guidelines. Statistical analysis was performed using 2-way ANOVA, with Dunnett’s multiple comparisons test. GraphPad Prism 8.4.3.

### Serology

#### Serum IgG Titers

Serology ELISAs were performed as described previously. Briefly, 96-well plates were coated with 300 ng/well of wild type (WT) proteins Hla, LukS, LukF, LukD, LukE, HlgA, HlgB, or HlgC or 100 ng/well of WT SEA, SEB, SEC1-3, SED, SEE, SHE, SEI, SEJ, TSST-1, or SpeB overnight at 4°C. Plates were washed and blocked with Starting Block (Thermo Fisher) for 1 h at room temperature (RT) followed by three washes. Plates were incubated for 1 h at RT with the test serum samples (diluted semi-log) and washed three times before applying goat anti-monkey IgG (H&L)-HRP (Horse Radish Peroxidase) at 0.1 µg/ml in starting block buffer. Plates were incubated for 1 h at RT, washed, and incubated with TMB (3,3′,5,5′-tetramethylbenzidine) to detect HRP activity for 30 min. 50 µl of stop solution (2N H_2_SO_4_) was added to all the wells. Optical density at 450nm was measured using a Versamax™ plate reader (Molecular Devices, CA). Data analysis for full dilution curves was performed using the SoftmaxPro software version 5.4.5 (Molecular Devices, CA) and ND_50_ values extrapolated.

#### Toxin Neutralizing Antibody Titers

##### Hla Toxin Neutralization Assay (TNA)

Rabbit blood was purchased from Colorado Serum Co. and used within 10 days of the date of blood drawn. The assay was performed as described previously (17). Briefly, whole blood was washed twice with PBS and re-suspended in PBS. In brief, 4% rabbit red blood cells (RBCs) were co-cultured with wild-type Hla ± serially diluted serum samples. Cells were centrifuged after 30 min and absorbance of supernatants determined at OD_416_nm.

##### PVL, HlgAB/CB, and LukED TNA

Leukocidin activity was determined based on cytotoxicity in dimethyl sulfoxide (DMSO) induced HL-60 cells (ATCC, Manassas, VA). The HL-60 cells were cultured for seven days in RPMI media supplemented with 10% fetal bovine serum (FBS) and 1.5% DMSO for optimal induction. Cell induction was confirmed by the expression of CD11b on induced versus non-induced cells using flow cytometry analysis. The cells were then harvested and washed with RPMI media containing 2% FBS. Serially two-fold diluted monkey serum pre-mixed with toxins PVL (4.16 nM), HlgCB (8.3 nM), HlgAB (20 nM), or LukED (80 nM) were then mixed with HL-60 derived neutrophils at a final density of 5 x 10^5^ cells/well, then incubated for 3 h at 37°C and 5% CO_2_. Cells alone and toxin alone were included as controls. Upon cell-supernatant incubation, this mixture was further incubated with 100 μg/ml of XTT with 1% electron coupling solution (Cell signaling) for 16 h and the cell viability was measured by colorimetric measurement at OD_470_nm (background subtracted at OD_690_nm).

##### Superantigen TNA

Commercially-sourced human peripheral blood mononuclear cells (PBMCs) were collected and isolated, using the Advarra Institutional Review Board (https://www.advarra.com/) with a peer-approved protocol, from heparinized blood of non-study de-identified healthy human donors by Ficoll gradient centrifugation as described elsewhere (18) and stored in liquid nitrogen until further use. All studies involving human samples were performed in accordance with the applicable guidelines and regulations. PBMCs were thawed, washed and resuspended in assay media to a density of 2×10^6^ viable cells/ml. 75 µl of this cell suspension with a viability of >95% was then added to a 96-well plate containing 75 µl of antibody/toxin pre-mixed at 1:1 as follows: semi-log dilutions of sera starting at 1:40 and a 0.1 ng/ml preparation of SEB, 1 ng/ml of either SEA, TSST-1, SEC-1, SEC-2, SEC-3, 3 ng/ml of SED, SEE, or SEH, 10 ng/ml of SEJ, and 30 ng/ml of SEI or SpeB. Wells containing medium with toxin only served as positive controls. The plates were incubated at 37°C in an atmosphere of 5% CO_2_-95% air for 48 h. Plates were centrifuged at 1,500 rpm for 10 min, culture supernatants harvested and IFNγ concentration (pg/ml) as a readout of superantigenicity was determined by ELISA. Plates were read at 450 nm using the Versamax plate reader. Data was analyzed using a 4-parameter (4PL) curve fit in XLFit (Microsoft). Toxin neutralizing activity was defined as the effective dilution of sera at the inflection point of the 4PL curve at which 50% of toxin activity was neutralized (ND_50_).

## Results

### Safety of Multicomponent Toxoid Vaccine

**Figure 1:**
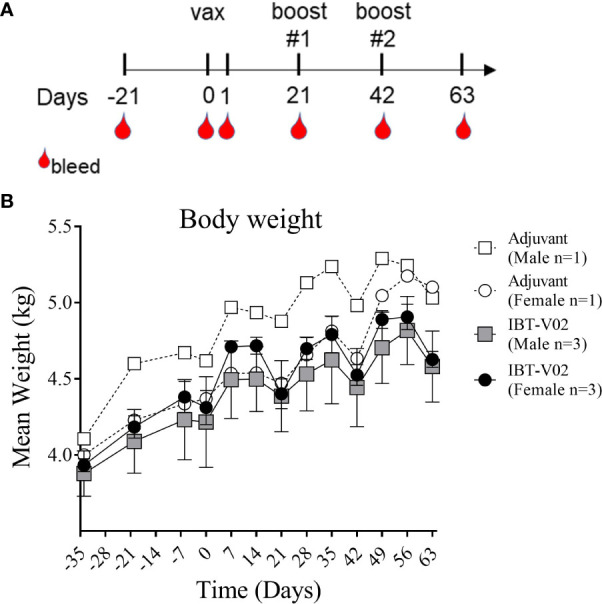
Study Design and Body weight change. Vaccination (Days 0, 21, 42) and blood collection schedule of NHPs **(A)**. Mean weight of non-human primates (NHPs) measured at indicated time points relative to study start on day of 1st vaccination (D0), and on a weekly basis thereafter until day 63 **(B)**. Data was analyzed with GraphPad PRISM v8.3.1.

Our primary objective in this study was to assess potential systemic toxicity and local reactogenicity of a 4-component staphylococcal toxoid vaccine in rhesus macaques when administered intramuscularly. Six NHPs (#201, #202, #203, #211, #212, #213) were immunized with a total of 400 µg of the 4-component vaccine (100 µg/antigen) in 2,000 µg Aluminum [4,000 µg Alhydrogel^®^ (Al(OH)_3_)] on days 0, 21 and 42. Two control NHPs (#101 and #111) were administered 4000µg Al(OH)_3_) alone. As displayed in **Figure 1A**, monkeys were bled 21 days prior to immunization to collect sera for baseline values. Additional bleeds were performed on days 1, 21 (prior to 1^st^ booster), 42 (prior to 2^nd^ booster) and 63. To evaluate potential toxicity of the 4-component vaccine the following primary endpoints were assessed: body weights, clinical observations, injection area assessment for edema and erythema following vaccine administration, and clinical pathology (serum chemistry and hematology).

#### Body Weights

Body weights were recorded as part of the quarantine period on days -34, -20, and -6 and continued to be recorded after study start on day 0 and on a weekly basis thereafter until day 63. Mean body weights of NHPs immunized with the 4-component vaccine or Al(OH)_3_ alone demonstrated a consistent increase over time with expected transitional decrease in body weights following overnight fasting for scheduled study events on days 0, 21, and 42 (**Figure 1B**).

#### Clinical Observations

NHPs were monitored twice daily for mortality and moribundity during the study period. There were no clinical observations that were considered related to the administration of the vaccine. Diarrhea, soft feces, emesis and low food consumption were observed sporadically in animals of both groups and were transient. There was no mortality during the study period.

#### Injection Site Observations

Injection sites were observed for erythema and edema prior to vaccination on days 0, 21, and 42 and at intervals of 1, 6, and 24 h after each vaccine administration. Injection sites were given scores for erythema ranging from 0- no redness to 4-severe redness and scores for edema ranging from 0- no swelling to 4- severe swelling (**Table S1**). No vaccine related injection site reactogenicity was noted. Three out of 8 NHPs (#111, #202, #212) had a score of 1- very slight or barely perceptible redness - for erythema observed at 6 h and 24 h (D1) after dose administration. However, on day 2 the score went back to 0 and initial redness was considered related to procedure of administration and not a response to the 4-component vaccine as the “very slight or barely perceptible redness” of 1.5 mm or less in size was observed in a control group animal (#111) as well as in animals (#202 and #212) receiving the vaccine.

#### Hematology

**Figure 2:**
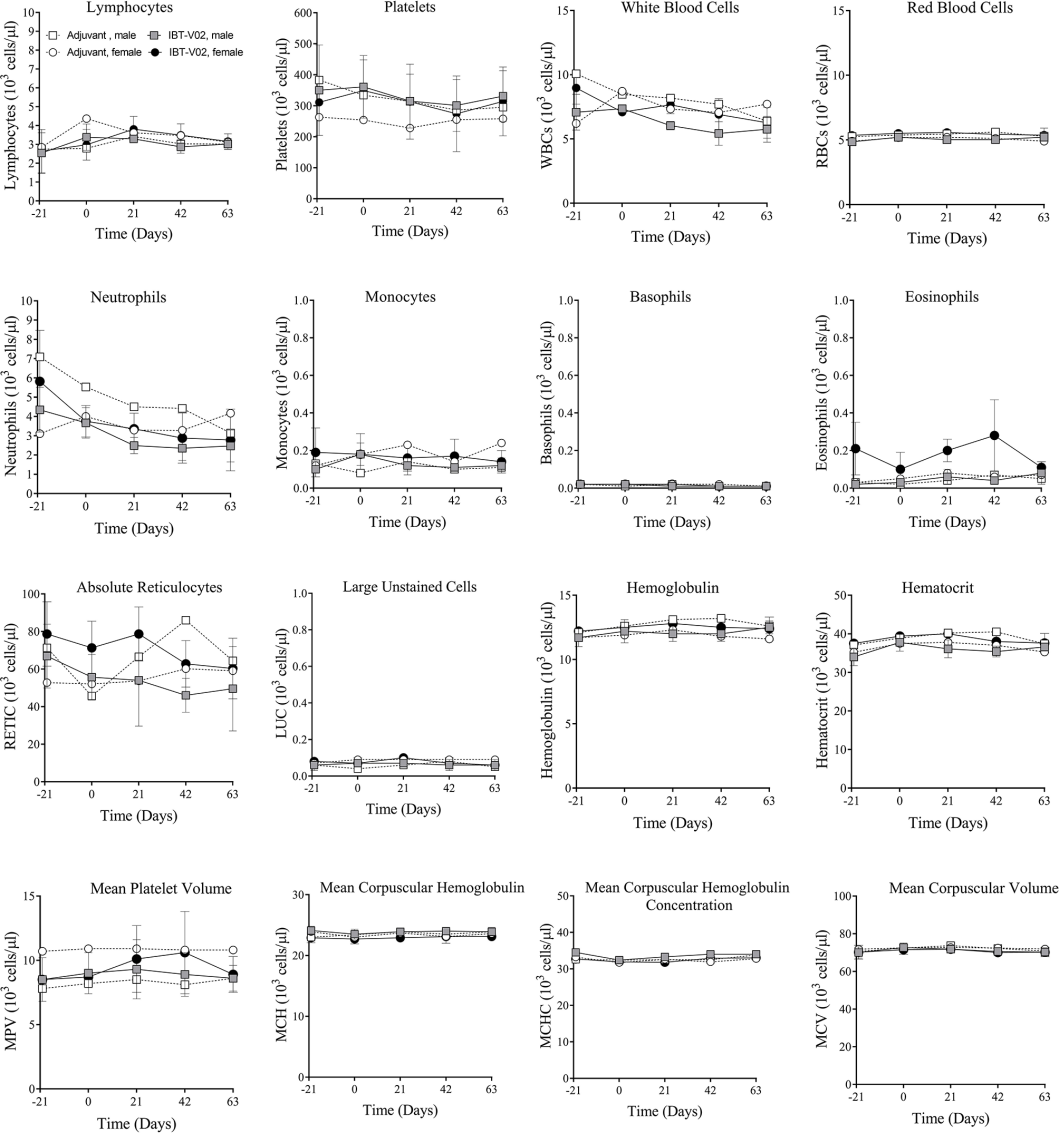
Hematology. Hematology data of non-human primates (NHPs) measured in serum samples collected on day 21 (D-21) and on day 0 (D0) prior to vaccination, and on days 1 (D1), 21 (D21), 42 (D42), and 63 (D63) after vaccination. Data was analyzed with GraphPad PRISM v8.3.1.

There were no vaccine-related findings noted in the hematology data. There were few changes noted in neutrophil counts, reticulocyte counts, platelet counts, and mean platelet volume of individual animals. However, due to the overall small magnitude of changes, the presence of these findings in only one animal and/or the presence prior to vaccine administration were considered incidental and related to biological variability rather than vaccine administration. Group mean hematology data for male and female NHPs are shown in **Figure 2** and individual hematology data are presented in **Table S2**.

#### Clinical Chemistry

**Figure 3:**
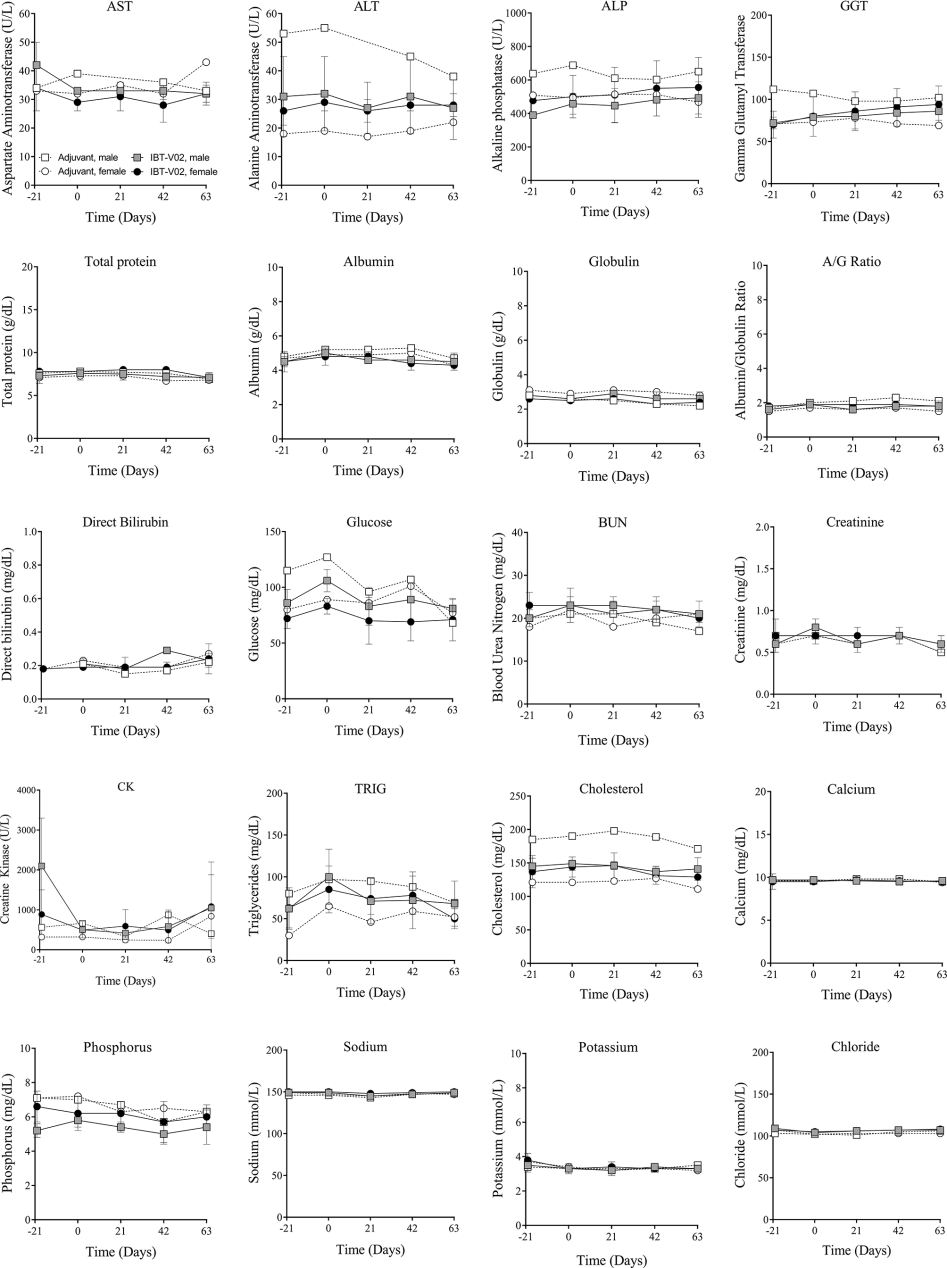
Serum chemistry. Chemistry values of non-human primates (NHPs) measured in serum samples collected at indicated timepoints before each vaccination on days 0 (D0), 21 (D21), and 42 (D42) and on day 63 (D63). Data was analyzed with GraphPad PRISM v8.3.1.

There were no vaccine-related findings noted in the serum chemistry data. All direct bilirubin and occasional total bilirubin values were below the reportable range. Total and direct bilirubin values near or below the lower limit of the reportable range are common in non-human primates. There were a few notable changes to CRP, LDH, ALP, triglycerides, and calcium levels. However, some of these findings were present at day -21 (prior to vaccination) and were considered incidental and related to biological variability rather than vaccine administration. Group mean serum chemistry data for male and female NHPs are presented in **Figure 3** and individual serum chemistry data are presented in **Table S3**. Taken together, hematology and serum chemistry parameters were within baseline biologic parameters previously reported in rhesus macaques (19, 20).

#### Serum Cytokines

**Figure 4:**
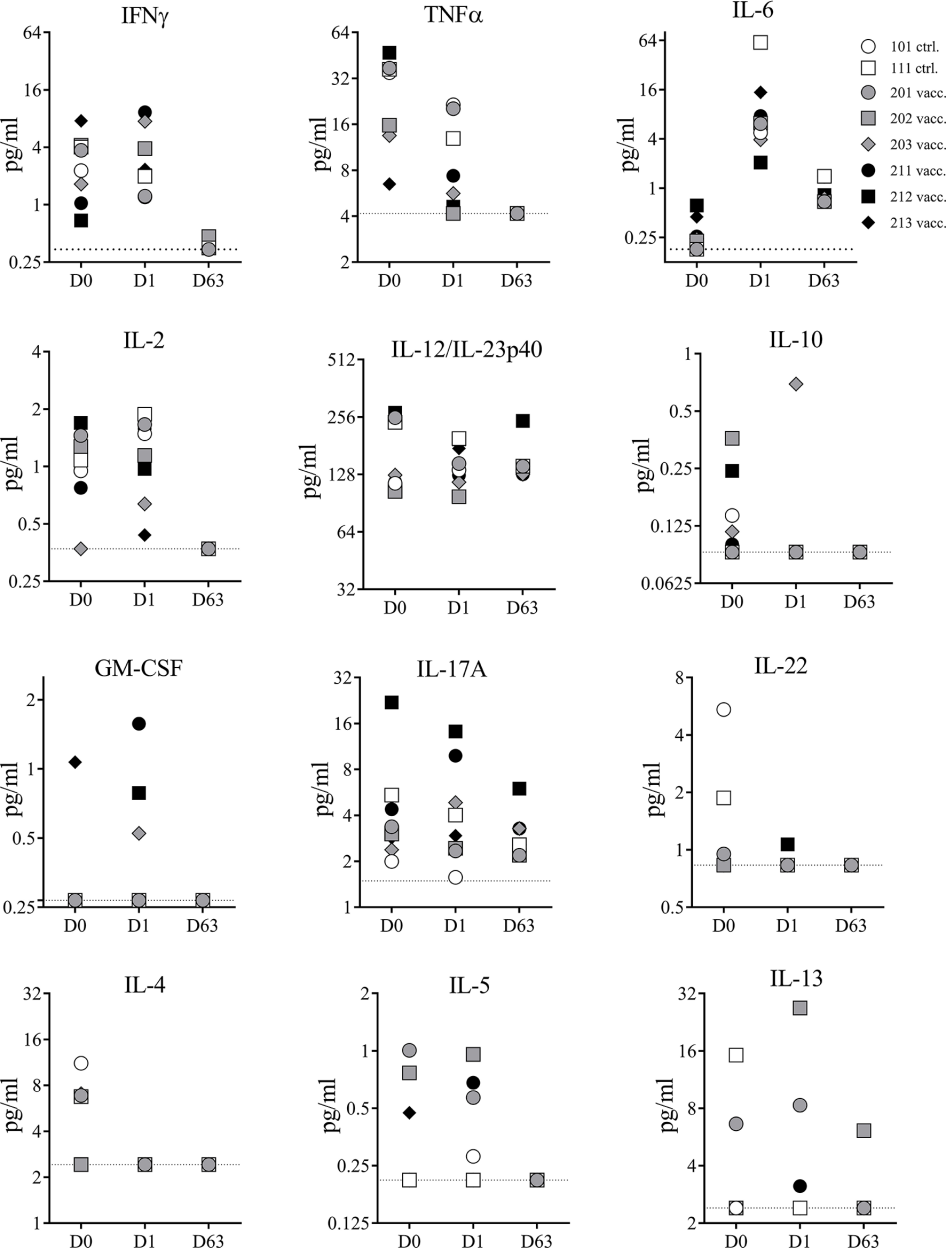
Serum cytokines. Cytokines measured in serum samples of non-human primates (NHPs) collected before vaccination (D0), and on days 1 (D1) and 63 (D63) after vaccination. NHPs receiving 4-component vaccine (vacc.) and NHPs receiving adjuvant only (ctrl.). Dotted lines reflect limit of detection for respective cytokine. Data was analyzed with GraphPad PRISM v8.3.1.

Superantigens can cause cytokine storm in humans and NHPs. To confirm the detoxified vaccine component TBA_225_ does not cause a cytokine storm in NHPs even when administered at a 10-fold higher dose than the projected human dose, the expression profile of 13 cytokines (IL-1β, IFNγ, TNFα, IL-2, IL-6, IL-12, IL-10, GM-CSF, IL-17-A, IL-22, IL-4, IL-5, and IL-13) circulating in serum was evaluated in samples collected prior to immunization on day 0, and after immunization on days 1 and 63 (**Figure 4**). There were no major changes in cytokine levels observed from D0 to D1 (1 day after first vaccination). Minor changes of increase or decrease in cytokine levels in individual animals were not vaccine-related and only transient as similar changes occurred in control animals and were not evident on day 63, suggesting that observed changes were related to the inflammatory properties or minor injection site reactogenicity of Al(OH)_3_. Three NHPs showed slightly elevated levels (<2 pg/ml) of GM-CSF on D1 but undetectable level on D63. No IL-1β was detectable at any given time point (data not shown). In summary, there were no acute adverse events induced by the 4-component vaccine. No sudden elevation in pro-inflammatory cytokines indicative of a cytokine storm caused by potential residual toxicity of attenuated superantigenic proteins was observed, confirming that the TBA_225_ component of the 4-component vaccine is non-toxic as it has been described previously (12) and well-tolerated in NHPs at a dose that is 10-fold higher than the projected human dose.

### Immunogenicity

As a secondary endpoint of our study, we assessed the immunogenicity of the 4-component vaccine.

#### IgG Titers of NHP Sera

**Figure 5:**
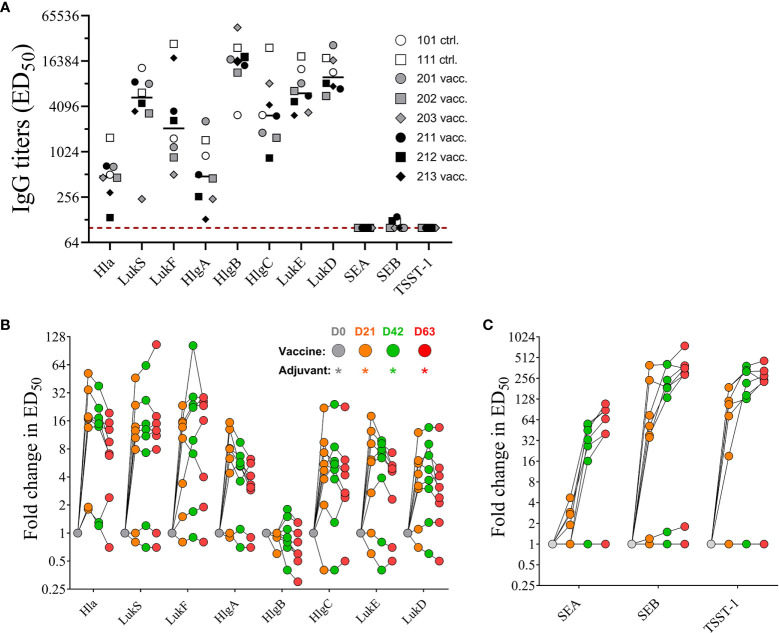
Serum IgG titers. Pre-existing serum titers against staphylococcal antigens in sera of non-human primates (NHPs) prior to vaccination. NHPs receiving 4-component vaccine (vacc.) and NHPs receiving adjuvant only (ctrl.) Dotted lines indicate limit of detection set at 100, the lowest serum dilution tested in the assay **(A)**. Fold increase of IgG titers of NHP sera collected on days 0, 21, 42, and 63 toward wildtype pore-forming toxin Hla, PVL subunits LukS and LukF, HlgAB/CB subunits HlgA, -B, and –C, and LukED subunits LukE and LukD **(B)** and superantigens SEA, SEB, and TSST-1 **(C)**. Data was analyzed with GraphPad PRISM v8.3.1.

Serum samples collected prior to first immunization on day 0 (baseline) and 21 days after each immunization on days 21, 42, and 63 were evaluated by ELISA for toxin specific IgG binding titers using the vaccine target antigens Hla, LukS-PV, and LukF-PV, and the superantigenic toxins SEA, SEB, and TSST-1. In addition to the direct target antigens, serum samples were also tested for cross-binding properties against closely related bi-component pore-forming toxin subunits HlgA and HlgC (S component), HlgB (F component) of gamma hemolysin as well as LukE (S subunit) and LukD (F subunit) of LukED (21). As previously reported, NHPs, similar to humans are natural hosts of *S. aureus* (22, 23) and thus are highly likely to have pre-existing serum antibody titers to staphylococcal antigens. Indeed, pre-vaccination titers toward tested pore forming toxin subunits were present in all eight monkeys to varying degrees, while titers toward SEA, SEB, and TSST-1 were either very low or below the limit of detection (< 1:100 serum dilution) in our assay (**Figure 5A**) concurring with previous findings that majority of *S. aureus* strains isolated from NHPs are superantigen gene negative (23). Post vaccination titers were expressed as fold change over baseline values. IgG titers for PFTs increased after only one immunization up to 52-fold for Hla, 46-fold for LukS, and 23-fold for LukF. With the exception of two monkeys that showed increase in titers for LukS (#202) and LukF (#203) booster immunizations did not elevate the titers against target PFTs any further (**Figure 5B**). A primary dose of the 4-component vaccine also increased titers for closely related PFTs HlgA, HlgC, LukE, and LukD but not HlgB (**Figure 5B**), but additional booster immunizations also showed no effect on elevating serum titers any further. Of note pre-existing titers against HlgB were highest among the animals. In contrast, while titers after 1^st^ vaccination increased for SEA up to 5-fold, for SEB up to 400-fold and for TSST-1 up to 190-fold from baseline titers on day 21, both booster immunizations induced further increase of IgG titers against all three superantigens SEA, SEB, and TSST-1 (**Figure 5C**). Two monkeys (#211, #212) showed no initial increase of SEA titers in day 21 samples and the overall low response of the remaining vaccinated animals to the primary vaccination suggested the necessity of a booster to induce toxin-specific titers.

#### Toxin Neutralizing Activity of NHP Sera

**Figure 6:**
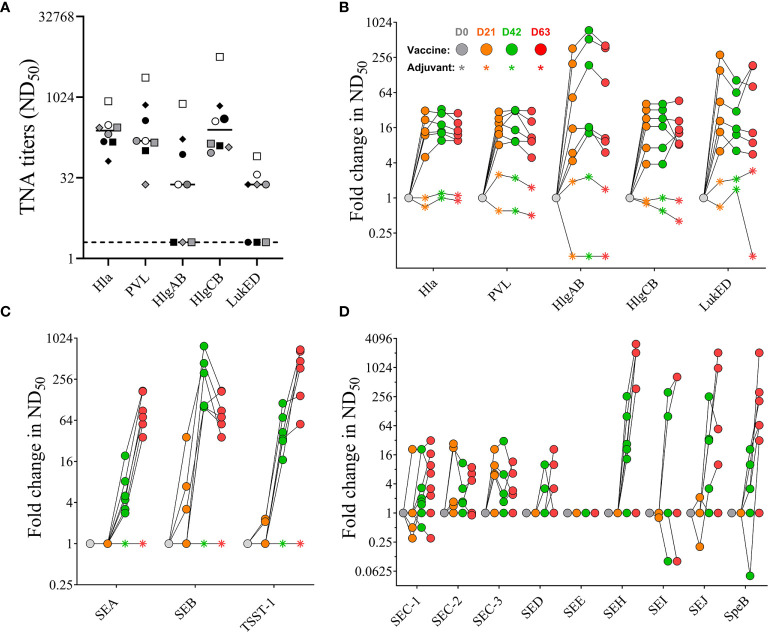
Serum toxin neutralizing antibody titers. Pre-existing neutralizing titers measured in serum samples before vaccination. Dotted lines indicate limit of detection set at 2, the lowest serum dilution tested in the assay **(A)**. Fold increase in Toxin neutralizing activity (TNA) and cross-neutralizing activity of non-human primate (NHP) sera collected on days 0, 21, 42, and 63 measured toward wildtype pore-forming toxins Hla, PVL, HlgAB, HlgCB, and LukED **(B)** and superantigens SEA, SEB, and TSST-1 **(C)**, as well as SEC-1-3, DED, SEE, SEH, SEI, and SEJ **(D)**. Colored symbols reflect indicated days post vaccination. Data was analyzed with GraphPad PRISM v8.3.1.

Sera were also tested for neutralizing activity against the target antigens Hla, PVL, SEA, SEB, and TSST-1 of the 4-component vaccine as well as for cross-neutralizing activity against the heterologous bi-component PFTs HlgAB, HlgCB, and LukED. No pre-existing neutralizing titers were detected against SEA, SEB, and TSST-1 (data not shown). With the exception of three out of eight monkeys that had no detectable neutralizing titers against HlgAB (#202, #203, #212) and LukED (#202, #211, 212) pre-vaccination sera from all animals showed neutralizing activity toward all tested PFTs (**Figure 6A**). Neutralizing titers toward Hla and PVL increased after primary vaccination but showed no further enhancement with booster vaccinations (**Figure 6B**). A single immunization with the 4-component vaccine induced maximal cross-neutralizing titers against heterologous PFTs (10–500 fold over background). However, a second vaccination only enhanced the cross neutralization to HlgAB (**Figure 6B**), where the highest fold change occurred in monkeys #202, #203, and #212, in which pre-existing titers were below detection limit (**Figure 6A**). In contrast to PFTs, neutralizing titers against superantigens SEA, SEB, and TSST-1 showed a major increase after two immunizations (titers tested on day 42 serum samples). While ND_50_ titers against SEA and TSST-1 increased with each booster and were highest on day 63, titers against SEB dropped by day 63 (**Figure 6C**). Immunization with the 4-component vaccine also elicited cross-neutralizing titers toward staphylococcal enterotoxin C (SEC) -1, -2, 3, SED, SEH, SEI, SEJ, and the heterologous streptococcal pyrogenic exotoxin B (SpeB) in most animals. No change in SEE titer was observed (**Figure 6D**).

Taken together, NHPs had pre-existing serum antibody titers recognizing staphylococcal antigens that could be enhanced not only in their binding but also in their neutralizing activity when immunized with the 4-component vaccine targeting Hla, PVL, SEA, SEB, and TSST-1. In addition, vaccination also increased cross-neutralizing activity toward heterologous BCPFTs HlgAB/CB and LukED, staphylococcal enterotoxins, as well as SpeB.

### Antibody and Cellular Immune Response After 3^rd^ Booster Immunization

**Figure 7:**
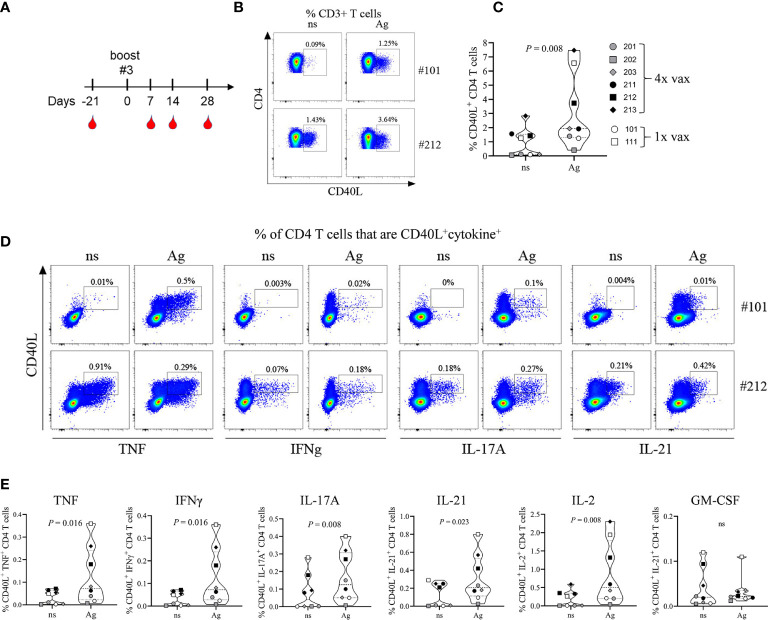
Immunophenotyping of vaccine-generated CD4 T cells. Additional blood collection and vaccination schedule for non-human primates (NHPs) before and after boost #3 **(A)**. Frequency of total CD40L^+^ CD4 T cells **(B, C)** or CD40L^+^ cytokine^+^ CD4 T cells **(D, E)** 14 days after primary vaccination (#101) or after boost #3 (#202). Representative plots **(B, D)** and summary data **(C, E)**. Non-stimulated (ns) and antigen stimulated (Ag) PBMCs. Data was analyzed with Flowjo v10.7 and GraphPad PRISM v8.3.1.

PBMCs that were collected during the 63-day time period (**Figure 1A**) and stored in liquid nitrogen were lost prior to use due to a mechanical failure of the storage unit. Study animals were still residing in the testing facility and were not yet assigned to another study. Therefore, after a resting period of 105 days all NHPs were re-enrolled for an extension of the study protocol allowing us to collect PBMCs for phenotypic and functional characterization of the cellular immune response toward the 4-component vaccine. As shown in **Figure 7A**, all animals previously assigned to this study protocol were bled 21 days prior to 3^rd^ booster immunization (corresponding to 168 days after the prime vaccination) of NHPs #201, #202, #203 (male) and #211, #212, #213 (female). In parallel the two adjuvant-only control NHPs (#101 and #111) were also immunized with the 4-component vaccine to allow characterizing the cellular response after a single immunization compared to four immunizations. PBMCs and plasma from all animals were collected 21 days prior to and 7, 14, and 28 days after the 3^rd^ booster vaccination.

#### IgG Titers of NHP Plasma

Plasma of all NHPs still showed IgG titers against the target antigens and closely related HlgA/B/C and LukE/D antigens prior to the 3^rd^ booster vaccination (**Figure S1A**), but showed a significant drop of LukS & F, LukE &D, and SEB and TSST-1 titers when compared to day 63 titers (**Figure S1B**). However, seven days following the 3^rd^ booster administration titers once again increased but dropped by day 28 against most pore-forming toxin antigens (**Figure S1C**). In contrast, titers against SEA, SEB and TSST-1 increased but were relatively stable over 28 days (**Figure S1D**).

#### T Cell Response Induced By 4-Component Vaccine

To identify vaccine-generated CD4 T cells PBMCs collected at different time points were re-stimulated *ex vivo* with the 4-component vaccine and after intracellular staining for TNF, IFNγ, IL-2, IL-17A, GM-CSF, and IL-21 were analyzed for the frequency of total cytokine producing CD4 T cells (**Figure S2**). All monkeys demonstrated an anamnestic response measured as increase in cytokine producing CD4 T cells *in vitro* toward the 4-component vaccine when compared to unstimulated controls. The magnitude of the response did not differ significantly from the primary (NHPs #101 and 111) to the quaternary immune response displayed by the animals that received a total of 4 immunizations (NHPs #201, 202, 203, 211, 212, 213). As five out of eight animals demonstrated a peak response on day 14 post vaccination CD4 T cells were further characterized for their functional phenotypes at this time point. Antigen-specific CD4 T cells were assessed by their expression of the activation marker CD40L (CD154) as described previously (24). *Ex vivo* re-stimulation of PBMCs with the 4-component vaccine (Ag) elicited an increased frequency of CD40L^+^ CD4 T cells when compared to non-stimulated (ns) controls in both animals receiving the vaccine once (**Figures 7B, C**, #101) or receiving it four times (**Figures 7B, C**, #212). While all animals responded to the antigen *ex vivo*, the frequency of CD40L^+^ CD4 T cells within CD3^+^ T cells varied among NHPs from as low as 0.4% (#202) to 7.5% (#213). Next, we compared the cytokine profile of CD4 T cells generated during primary and during quaternary response to the vaccine. PBMCs of NHPs that were vaccinated once (#101) or vaccinated four times (#212) were re-stimulated *ex vivo* with the 4-component vaccine. CD40L^+^ CD4 T cells produced cytokines characteristic of the Th1 (TNF, IFNγ, IL-2) and the Th17 (IL-17A, IL-21) subsets as shown in the example FACS plots in **Figure 7D** and summary plots in **Figure 7E**. Although GM-CSF was detectable, there was no antigen-specific increase in GM-CSF producing CD4 T cells.

#### Cellular Cytokines Induced By 4-Component Vaccine

**Figure 8:**
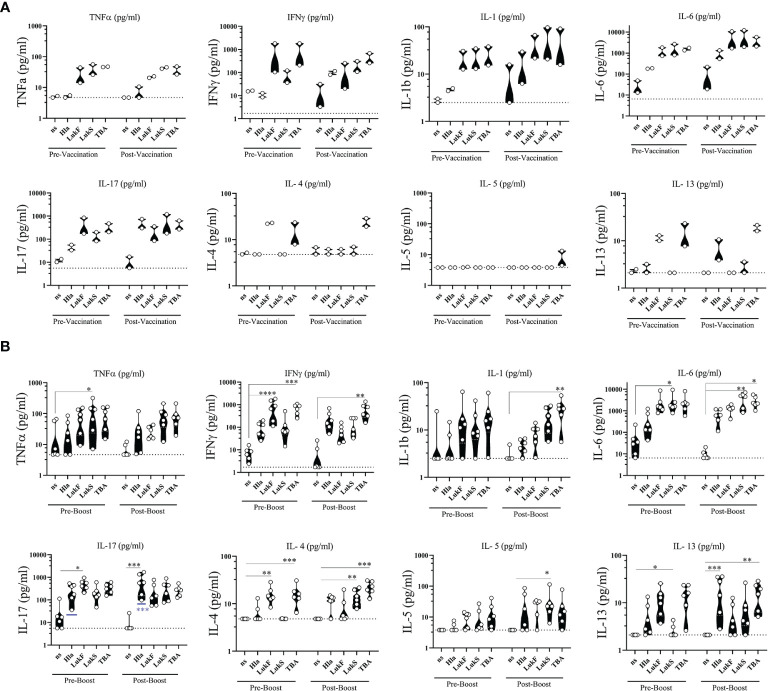
*Ex vivo* cytokine release. Cytokines released from PBMCs of non-human primates (NHPs) in response to stimulation with individual vaccine components Hla_H35LH48L_ (Hla), LukF_mut1_ (LukF), LukS_mut9_ (LukS), and TBA_225_ (TBA). **(A)** PBMCs were collected prior to (Pre-Vaccination) and 14 days after 1^st^ vaccination (Post-Vaccination). **(B)** PBMCs were collected prior to (Pre-Boost) and 14 days after 3^rd^ booster vaccination (Post-Boost). Controls were left non-stimulated (ns). Shown are violin plots with individual data points. Limit of detection is shown as dashed grey lines. *<0.05; **<0.01; ***<0.001; ****<0.0001. Data was analyzed with GraphPad PRISM v8.3.1.

To complement the functional phenotyping of vaccine generated CD4 T cells by flow cytometry we stimulated PBMCs with the individual vaccine components Hla_H35L/H48L_, LukF_mut1_, LukS_mut9_, and TBA_225_ for 24 h and performed multiplex assays on the culture supernatants to quantify a panel of 8 cytokines (TNFα, IFNγ, IL-1, IL-6, IL-17, IL-4, IL-5, IL-13). Non-stimulated (ns) PBMCs were used as controls for baseline levels. The cytokine profile during primary response was evaluated in NHPs #101 and #111 two weeks after vaccination (Post-Vaccination) and compared to Pre-vaccination values. Interestingly, we observed that PBMCs of NHPs collected prior to vaccination demonstrated a similar cytokine response to individual vaccine components as PBMCs collected after vaccination, with a few exceptions where cytokines IFNγ, IL-6, and IL-17 increased in response to Hla after vaccination (**Figure 8A**). The cytokine profile during the quaternary response was evaluated in 4x vaccinated NHPs two weeks after the 3^rd^ booster immunization compared to pre-boost (3x vaccinated) samples. Pre-boost and post boost cytokine response to the individual components was similar in pattern and while each component was able to induce cytokine production by PBMCs, the most pronounced cytokines were IL-6 followed by IL-17 and IFNγ (**Figure 8B**). There was also a significant increase in IL-17 from three vaccinations to four vaccinations in response to Hla. Taken together, immunization with the 4-component vaccine in Alhydrogel generated antigen-specific CD4 T cells of a Th1/Th17 phenotype.

## Discussion

Many attempts over the past two decades to develop an effective staphylococcal vaccine have either failed or in one case caused detrimental outcomes upon infection (4, 25). While all these vaccine candidates targeted surface antigens of *S. aureus*, emerging findings on the pathogenesis of *S. aureus* and clinical and epidemiological studies point to the importance of staphylococcal toxins as vaccine targets (26). *S. aureus* produces a wide range of cytolytic and superantigenic toxins that have largely evolved to target specific human immune receptors. Therefore, the choice of animal model for evaluation of safety and efficacy of *S. aureus* toxoid vaccine candidates is critically important. For example, the affinity of all SAgs for human MHC class II molecules, the primary receptor for all SAgs, is far greater than for the mouse counterparts leading to poor response of mice to SAgs (27). Here we elected to evaluate the safety and immunogenicity of a four-component toxoid vaccine candidate in rhesus macaques, known to be highly sensitive to SAgs and multiple cytolytic toxins of *S. aureus* (14–16). This study shows that the vaccine is safe, well tolerated, and highly immunogenic inducing neutralizing antibodies and specific memory T cell responses in macaques.

Facing the increasing emergence of highly virulent, multi-drug resistant *S. aureus* strains an effective vaccine is of utmost importance. In the past two decades intensive research has focused on the paradigm of targeting surface antigens to generate opsonizing antibodies that enhance phagocytic uptake and clearance of the pathogen. This approach while validated by capsular polysaccharide (CP) vaccines developed against encapsulated bacteria like *Streptococcus pneumoniae*, *Hemophilus influenzae type B* or *Neisseria meningitidis* and *Salmonella typhi* (28–32) has not proven clinically successful against *S. aureus* (25) suggesting that capsular polysaccharides might not be the major driver of virulence in *S. aureus* pathogenesis. This is supported by the fact that although clinical *S. aureus* isolates predominantly express CP type 5 or 8 (CP5, CP8) highly virulent strains like USA300 and USA500 lack capsule due to mutations in their Cap5 gene (33). Similarly, attempts to generate opsonizing antibodies against other surface associated antigens or the use of surface targeted monoclonal antibodies have not proven to be viable options for this pathogen (4, 34–37). Our approach of targeting secreted toxin antigens is encouraged by the compelling evidence that the presence of anti-toxin antibodies, in particular directed against pore forming toxins (PFTs) and superantigens (SAgs) strongly correlates with a protective outcome in a wide range of infections caused by *S. aureus* as recently summarized in an excellent review by Miller et al. (26).

We have previously reported on the development of rationally designed toxoids specifically targeting cytolytic PFTs and SAgs with the goal to generate toxin-neutralizing rather than pathogen-opsonizing serum antibodies (10–12, 17, 38–40). Alpha hemolysin (Hla) is a widely expressed single component cytolysin with broad range of lytic activity in rodents, NHPs, and humans toward immune cells, epithelial cells and keratinocytes, as well as erythrocytes (41). The bicomponent family of cytolysins include the closely related PVL, HlgAB, HlgCB, and LukED, as well as phylogenetically more distant LukAB (42). These toxins consist of two subunits S and F and primarily target immune cells of critical importance for defense against *S. aureus* including neutrophils, macrophages, monocytes, dendritic cells, as well at T cells (43). Superantigens constitute a large family including multiple staphylococcal enterotoxins and TSST-1 (27). SAgs cross-link the T cell receptor with MHC Class II and activate up to 30% of T cells, leading to a cytokine and chemokine storm and massive T-cell proliferation (44). These events can culminate in Toxic Shock Syndrome (TSS). At non-TSS inducing concentrations, SAgs impact *S. aureus* virulence through induction of a local excessive inflammatory response (45, 46), drive pathologic Th2 responses during infections (47–50), and cause T cell anergy (51) and T cell dependent B cell apoptosis (52), further impairing adaptive immune responses to *S. aureus*. Furthermore, SAgs, in particular SEB, can have devastating effects at miniscule amounts when used as biowarfare agents resulting in organ failure and death, leading to efforts to develop and use these toxins as biowarfare agents (53).

The 4-component vaccine candidate presented here is composed of mutants of the S and F subunits of PVL (LukS_mut9_ and LukF_mut1_) (10) and a double mutant of Hla (Hla_H35L/H48L_) (11). These toxoids are engineered to lack the ability to oligomerize upon interaction with the plasma membrane and therefore fully attenuated. Our serological studies have shown that targeting SEA, SEB, and TSST-1 can provide a broad neutralizing response providing a rationale for generation of the fusion toxoid TBA_225_ (12). While we have previously demonstrated full attenuation of TBA_225_ *in vitro, (* 12) it was particularly important to ensure the safety of this toxoid in a highly sensitive model such as macaques. Vaccinated animals showed weight gain similar to the controls during the course of study (**Figure 1**) and no abnormalities in blood chemistry and hematology analysis were observed (**Figures 2** and **3**). Local reactogenicity observations that were made in three animals were considered related to administration procedure and not a response to the vaccine. The “very slight or barely perceptible redness” at the injection site was observed in both a control group animal (#111) as well as two vaccine group animals (#202, #212) and resolved within two days of injection.

Superantigens are of particular concern with respect to cytokine storm as a result of polyclonal T cell activation (44). While we had previously demonstrated the lack of superantigenic activity of TBA_225_ (12)as well as safety of one of the components of TBA_225_ fusion protein (STEBVax) in a clinical dose escalation phase 1 study (40) in this study we further examined the acute cytokine response in NHPs vaccinated with the 4-component vaccine. Twenty-four hours after vaccination the circulating levels of inflammatory cytokines were very low to undetectable and, importantly, did not differ between the vaccinated and control animals, indicating lack of *in vivo* superantigenic activity of the vaccine. Given the high sensitivity of NHPs to SAgs, these data clearly justify evaluation of the vaccine in humans.

Macaques are sensitive to toxic effects of Hla, with doses of 100 mg/Kg leading to acute toxicity including cytokine induction, drop in platelet counts, hypotension, and electrocardiogram abnormalities and even death when treated with 200 mg/Kg (14). Hla is also known to increase meningeal permeability in monkeys (54). Hla_H35L_ (single mutant) has been previously shown to be safe in two phase I clinical trials (55, 56). Our study further provides formal evidence for the safety of Hla_H35LH48L_ (double mutant) in a highly relevant model. In contrast to Hla and SAgs, macaques are not sensitive to PVL (57). Thus, a limitation of our study is that it does not address the safety of the PVL components of the vaccine with respect to any potential residual activity of these components toward immune cells.

The 4-component vaccine generated a robust total (IgG) and neutralizing antibody response toward each individual target antigen. Serum antibodies not only showed neutralizing activity toward vaccine targets but also cross-neutralizing activity toward structurally related staphylococcal antigens within the families of bicomponent pore forming toxins as well as superantigens, an important characteristic making this vaccine applicable against a wide range of *S. aureus* strains.

NHPs, similar to humans are natural hosts of *S. aureus* and have detectable pre-existing serum antibody titers that are specific for a wide range of staphylococcal antigens (23, 58, 59). As presented, NHPs exhibited pre-existing neutralizing antibodies for almost all tested antigens and a single vaccination was sufficient to enhance these neutralizing titers, suggesting that a single vaccination in humans could also be sufficient to elicit a robust toxin neutralizing antibody response. Interestingly, no IgG titers were detected against the three SAgs SEA, SEB, and TSST-1 in pre-vaccination sera of NHPs, which could be explained by a recent report showing that a majority of tested NHP isolates were negative for SAgs (23). Importantly, for the majority of tested pore-forming toxins a single dose of the vaccine was sufficient for achieving the maximal neutralizing response (**Figure 6B**). However, for SEA, SEB, and TSST-1 two-three doses were required. The rapid response indicates that the vaccine induces anamnestic responses.

While waning of antibody titers over time is not unusual and in fact expected as the antigen gets cleared it is noteworthy that we observed a drop in titers after a booster vaccination. However, the observed decline in titers against pore-forming toxins after the 2^nd^ booster immunization (**Figures 5B**, **6B**) as well as after the resting period of 100 days (**Figure S1B**) was unexpected and needs to be further investigated. Since the primary goal of this study was to determine the safety of the vaccine we used a high vaccination dose to enable observation of any eventual toxicity. We cannot rule out that the observed phenomenon relates to the extremely high vaccine dose used in this study. We are currently investigating the impact of dose and vaccination schedule on the duration of immunity in NHPs.

Immune defects that pre-dispose humans to *S. aureus* disease have helped reveal the pivotal role of CD4 T cells during *S. aureus* infections. Patients with autosomal dominant hyper IgE syndrome due to STAT3 dysfunction resulting in impaired Th17 generation, are prone to develop cutaneous and pulmonary *S. aureus* infections (60–62). Similarly, HIV patients with decreased CD4 T cell counts are at increased risk to develop skin as well as systemic *S. aureus* infections (63, 64). In HIV patients the increased likelihood of skin infections was shown to correlate with the depletion of Th17 cells during early course of disease (65), and more recently the increased susceptibility to skin infections has been also associated with an impaired Th1 response (66). Th1 and Th17 cells mediate their effector functions through the production of their signature cytokines IFNγ and IL-17, respectively. These cytokines facilitate activation of macrophages and mobilization and recruitment of neutrophils (67) to sites of infection and consequently enhance phagocytic activity and bacterial clearance. The protective role of vaccine-generated Th1 and Th17 subsets and their downstream effects has also been demonstrated in pre-clinical experimental settings (68–72), however to date none of the vaccine candidates have been translated into clinical success. We and others have recently reported that vaccination with cell surface antigens either purified or in form of a whole cell vaccine preparation can enhance pathogenesis of *S. aureus* disease. While underlying cell-mediated immune responses are unknown for the former study, a vaccine generated dominating Th1/IFNγ response was responsible for the deleterious outcome in the latter (6, 7). While the importance of vaccine generated adaptive immune responses toward *S. aureus* infections has been acknowledged, the choice of target antigen and the type of generated CD4 T cells that is not biased heavily toward one or the other lineage deserves equal attention. Here, we present that immunization with the 4-component vaccine elicits a well-balanced CD4 T cell response generating antigen-specific IFNγ^+^ and IL-17^+^ as well as IL-21^+^ CD4 T cells. IL-21 is a pleiotropic cytokine that is mainly produced by T helper cells and is well established to support the generation and differentiation of B cells into antibody secreting plasma cells with the help of IL-6 (73–76). Hence, the presence of IL-21 producing T helper cells is in line with the specific toxin-neutralizing antibodies generated after immunization with the 4-component vaccine.

Multiplex analysis of supernatants from antigen-stimulated PBMCs revealed that each individual component of the 4-component vaccine efficiently induced cytokine release when compared to non-stimulated controls. Lower levels of Th2 cytokines IL-4, IL-5, and IL-13 but preferential release of Th1 and Th17 cytokines IFNγ and IL-17 measured in culture supernatants was in accordance with the functional phenotype of the antigen-specific CD4 T cells assessed by flow cytometry. The presence of high IL-6 levels in PBMC supernatants induces IL-21 upregulation in T helper cells which in turn can support the differentiation of B cells into antibody-secreting plasma cells. These data support the finding that the 4-component vaccine is skewing toward a Th1/Th17 and away from a Th2 type response. This could be an encouraging characteristic in a potential staphylococcal vaccine with broad application, as some inflammatory skin diseases like atopic dermatitis (AD) have been associated with type 2 inflammatory cytokines creating a milieu that is conducive to bacterial growth. Patients with AD show increased colonization and infection with *S. aureus* (77–79). Staphylococcal superantigens are likely a major factor in the Th2 type inflammatory response seen in AD and disease severity correlates with levels of SAg expression levels (80, 81). Furthermore, SAgs also facilitate the epithelial presentation of allergens to Th2 cells  (82). Adhesion molecules that bind to *S. aureus* such as fibronectin and laminin are upregulated as a result of the Th2 cytokine IL-4 released in the inflammatory environment of skin promoting disease pathogenesis (83) and together with IL-13 can hamper Th17 induced anti-microbial peptides produced by keratinocytes (84). Thus, neutralization of superantigens by vaccine-generated antibodies and polarization toward Th1 and Th17 differentiation is expected to change the immunological environment to unfavorable conditions for colonization and infection.

Taken together, our study reports on a 4-component vaccine that is safe and engages both, the humoral arm and the cellular arm of the immune system, eliciting toxin neutralizing antibodies and driving a balanced Th1/Th17 immune response, respectively. While the humoral component can reduce tissue damage resulting from PFTs and limit non-specific inflammation resulting from SAgs, the cellular component can activate downstream mechanisms of the innate immune system *via* cytokines like IL-17 and IFNγ to enhance phagocytic uptake and bacterial clearance.

Hence, we believe that targeting secreted toxin antigens could be the next-generation approach for staphylococcal vaccines if also proven to provide efficacy in humans.

## Data Availability Statement

The original contributions presented in the study are included in the article/**Supplementary Material**. Further inquiries can be directed to the corresponding author.

## Ethics Statement

The animal study was reviewed and approved by Battelle IACUC (study #103468A) - West Jefferson, OH 43162.

## Author Contributions

AV, HK, DB, and MA designed experiments. AV, GL, EC, KK, NL, TK, RA, and HK carried out experiments and analyzed data. DB, MA, FH, and HK interpreted data. KE, DK, and TR performed safety and immunogenicity in NHPs. AV, MA, and HK wrote manuscript. Funding acquisition, MA. All authors contributed to the article and approved the submitted version.

## Funding

KK, NL, and DB were supported by the Intramural Research Program of NIAID/NIH. This project has been funded in whole or in part with Federal funds from the National Institute of Allergy and Infectious Diseases, National Institutes of Health, Department of Health and Human Services, under NIAID’s Preclinical Services Contract No. HHSN272201200003I/HHSN27200022, supported under NIAID grant R01-AI111205. Research reported in this publication is supported by the Cooperative Agreement Number IDSEP160030 from ASPR/BARDA and by awards from Wellcome Trust, Germany’s Federal Ministry of Education and Research, the UK Global Antimicrobial Resistance Innovation Fund (GAMRIF) and the Bill & Melinda Gates Foundation, as administrated by CARB-X. The content is solely the responsibility of the authors and does not necessarily represent the official views of the Department of Health and Human Services Office of the Assistant Secretary for Preparedness and Response, other funders, or CARB-X.

## Conflict of Interest

MA and HK have stocks and RA and FH have stock options in Integrated Biotherapeutics Inc.

The remaining authors declare that the research was conducted in the absence of any commercial or financial relationships that could be construed as a potential conflict of interest.
