# Supplementary material for: Safety and Immunogenicity of a 4-Component Toxoid-Based Staphylococcus aureus Vaccine in Rhesus Macaques
Source: Front Immunol. 2021 Feb 25;12:621754. doi: 10.3389/fimmu.2021.621754 (PMC7947289; doi:10.3389/fimmu.2021.621754)
Supplement: Supplementary file 1 [file DataSheet_1.pdf]

	Score	Symptom
Erythema	0	No redness
	1	Very slight or barely perceptible redness
	2	Well defined redness
	3	Moderate to severe redness
	4	Severe redness (beet redness) to eschar formations (injuries in depth)
Edema	0	No swelling
	1	Very slight or barely perceptible swelling
	2	Slight swelling (edges of are well-defined by definite raising)
	3	Moderate swelling (edges raised approximately 1mm)
	4	Severe swelling (raised morea than 1mm and extending beyond the exposure area)

Table S1: Injection site observation. Scoring table for Erythema and Edema at the injection site.

Day -21

NHP	Red blood cells (x10^6 cell/ul)	Hemoglobin (g/dL)	Hematocrit (%)	MCV (fL)	MCH (pg)	MCHC (g/dL)	RETIC (x10^3 cells/µL)	White Blood Cells (x10^3 cells/µL)	Lymphocytes (x10^3 cells/µL)	Neutrophils (x10^3 cells/µL)	Monocytes (x10^3 cells/µL)	Eosinophils (x10^3 cells/µL)	Basophils (x10^3 cells/µL)	LUC (x10^3 cells/µL)	Platelets (x10^3 cells/µL)	MPV (fL)	Group	Sex
101	5.26	12.1	37.1	70.6	23	32.6	71.3	10.07	2.74	7.09	0.13	0.03	0.02	0.06	383	7.8	Adjuvant	M
111	4.90	11.7	35.2	71.8	23.9	33.2	52.8	6.20	2.85	3.11	0.12	0.03	0.02	0.07	263	10.7	Adjuvant	F
201	4.66	11.5	33.8	72.4	24.7	34.1	77.3	6.96	1.47	5.33	0.10	0.01	0.01	0.03	265	7.8	Vaccine	M
202	5.12	12.5	36.3	70.9	24.4	34.5	76	5.75	2.51	3.07	0.06	0.03	0.02	0.07	518	7.3	Vaccine	M
203	4.77	11.1	31.8	66.7	23.3	34.9	47.3	8.53	3.63	4.64	0.14	0.02	0.02	0.09	266	10.5	Vaccine	M
211	5.12	11.9	37.3	72.8	23.3	32	78.2	10.23	1.42	8.54	0.12	0.05	0.02	0.07	317	8.7	Vaccine	F
212	5.61	12.4	37.0	66	22.0	33.4	61.8	7.71	3.69	3.23	0.34	0.30	0.03	0.1	356	8.1	Vaccine	F
213	5.32	12.4	38.2	71.7	23.4	32.6	96.2	8.97	2.82	5.66	0.11	0.29	0.02	0.06	261	8.7	Vaccine	F

Day 0

NHP	Red blood cells (x10^6 cell/ul)	Hemoglobin (g/dL)	Hematocrit (%)	MCV (fL)	MCH (pg)	MCHC (g/dL)	RETIC (x10^3 cells/µL)	White Blood Cells (x10^3 cells/µL)	Lymphocytes (x10^3 cells/µL)	Neutrophils (x10^3 cells/µL)	Monocytes (x10^3 cells/µL)	Eosinophils (x10^3 cells/µL)	Basophils (x10^3 cells/µL)	LUC (x10^3 cells/µL)	Platelets (x10^3 cells/µL)	MPV (fL)	Group	Sex
101	5.37	12.6	38.9	72.4	23.4	32.4	45.7	8.47	2.79	5.53	0.08	0.02	0.01	0.04	334	8.2	Adjuvant	M
111	5.18	11.9	37.5	72.5	23.0	31.7	52.1	8.71	4.37	4.00	0.18	0.05	0.02	0.09	254	10.9	Adjuvant	F
201	5.13	12.3	38.5	75	24.0	31.9	63.6	7.53	2.72	4.49	0.23	0.02	0.02	0.05	272	8.7	Vaccine	M
202	5.46	13.1	39.7	72.7	24.0	33.0	61.8	7.16	3.3	3.61	0.11	0.02	0.02	0.09	470	7.6	Vaccine	M
203	5.01	11.3	35.3	70.3	22.6	32.2	41.7	7.36	4.11	2.29	0.20	0.04	0.02	0.08	340	10.7	Vaccine	M
211	5.29	12.1	38.3	72.4	22.9	31.7	57.8	6.76	2.32	4.24	0.09	0.03	0.02	0.07	328	8.7	Vaccine	F
212	5.76	12.6	39.5	68.5	21.9	32	86.1	7.31	3.9	2.8	0.30	0.20	0.02	0.10	457	8.7	Vaccine	F
213	5.49	12.9	40.4	73.5	23.4	31.9	69.9	7.21	2.73	4.2	0.14	0.07	0.01	0.05	262	8.7	Vaccine	F

Day 21

NHP	Red blood cells (x10^6 cell/ul)	Hemoglobin (g/dL)	Hematocrit (%)	MCV (fL)	MCH (pg)	MCHC (g/dL)	RETIC (x10^3 cells/µL)	White Blood Cells (x10^3 cells/µL)	Lymphocytes (x10^3 cells/µL)	Neutrophils (x10^3 cells/µL)	Monocytes (x10^3 cells/µL)	Eosinophils (x10^3 cells/µL)	Basophils (x10^3 cells/µL)	LUC (x10^3 cells/µL)	Platelets (x10^3 cells/µL)	MPV (fL)	Group	Sex
101	5.46	13.1	40.2	73.7	23.9	32.5	66.6	8.18	3.42	4.50	0.14	0.04	0.02	0.06	314	8.5	Adjuvant	M
111	5.20	12.3	37.8	72.7	23.7	32.6	53.7	7.33	3.62	3.29	0.23	0.08	0.02	0.09	228	10.9	Adjuvant	F
201	5.17	12.4	38.3	74.1	24.0	32.4	82.1	5.66	3.22	2.08	0.17	0.10	0.02	0.06	263	9.0	Vaccine	M
202	5.10	12.3	36.1	70.9	24.1	34.1	38.9	6.28	3.15	2.92	0.08	0.04	0.01	0.08	416	7.2	Vaccine	M
203	4.80	11.3	33.8	70.5	23.6	33.4	41	6.18	3.49	2.46	0.12	0.03	0.01	0.07	266	11.7	Vaccine	M
211	5.46	12.6	39.6	72.7	23.2	31.7	73.8	8.13	4.59	3.00	0.13	0.27	0.02	0.11	314	8.7	Vaccine	F
212	5.92	13.1	41.1	69.4	22.1	31.8	67.4	6.87	3.53	2.79	0.24	0.18	0.02	0.11	433	8.5	Vaccine	F
213	5.36	12.6	39.3	73.4	23.4	31.9	94.9	7.99	3.32	4.29	0.12	0.15	0.01	0.08	192	13.1	Vaccine	F

Day 42

NHP	Red blood cells (x10^6 cell/ul)	Hemoglobin (g/dL)	Hematocrit (%)	MCV (fL)	MCH (pg)	MCHC (g/dL)	RETIC (x10^3 cells/µL)	White Blood Cells (x10^3 cells/µL)	Lymphocytes (x10^3 cells/µL)	Neutrophils (x10^3 cells/µL)	Monocytes (x10^3 cells/µL)	Eosinophils (x10^3 cells/µL)	Basophils (x10^3 cells/µL)	LUC (x10^3 cells/µL)	Platelets (x10^3 cells/µL)	MPV (fL)	Group	Sex
101	5.6	13.2	40.5	72.2	23.5	32.5	86.0	7.7	3.03	4.42	0.10	0.07	0.01	0.08	286	8.1	Adjuvant	M
111	5.11	11.8	37.0	72.4	23.1	31.9	60.2	7.06	3.47	3.28	0.14	0.06	0.02	0.09	255	10.8	Adjuvant	F
201	5.04	12.2	36.5	72.4	24.2	33.5	56.1	4.37	2.56	1.63	0.12	0.03	0.01	0.03	218	9.2	Vaccine	M
202	5.17	12.5	36	69.6	24.1	34.6	42.7	5.85	2.83	2.81	0.08	0.04	0.01	0.08	385	7.1	Vaccine	M
203	4.85	11.4	33.8	69.8	23.6	33.8	39.1	6.07	3.23	2.6	0.12	0.06	0.01	0.06	300	10.4	Vaccine	M
211	5.16	11.8	36.2	70.1	22.9	32.7	74.6	5.47	3.28	1.76	0.09	0.28	0.01	0.04	290	8.2	Vaccine	F
212	5.91	13.0	40.0	67.7	22.1	32.6	49.9	7.6	4.16	2.59	0.26	0.47	0.02	0.10	387	9.3	Vaccine	F
213	5.22	12.6	37.8	72.3	24.2	33.4	63.8	7.64	3.02	4.3	0.15	0.09	0.01	0.07	144	14.2	Vaccine	F

Day 63

NHP	Red blood cells (x10^6 cell/ul)	Hemoglobin (g/dL)	Hematocrit (%)	MCV (fL)	MCH (pg)	MCHC (g/dL)	RETIC (x10^3 cells/µL)	White Blood Cells (x10^3 cells/µL)	Lymphocytes (x10^3 cells/µL)	Neutrophils (x10^3 cells/µL)	Monocytes (x10^3 cells/µL)	Eosinophils (x10^3 cells/µL)	Basophils (x10^3 cells/µL)	LUC (x10^3 cells/µL)	Platelets (x10^3 cells/µL)	MPV (fL)	Group	Sex
101	5.28	12.6	37.4	70.8	23.9	33.8	64.4	6.39	3.02	3.14	0.11	0.05	0.01	0.05	295	8.6	Adjuvant	M
111	4.9	11.6	35.3	71.9	23.6	32.8	59.1	7.71	3.12	4.17	0.24	0.07	0.01	0.09	258	10.8	Adjuvant	F
201	5.01	12.2	36.5	72.8	24.4	33.4	75.1	4.72	2.7	1.71	0.12	0.15	0.01	0.03	260	9.1	Vaccine	M
202	5.36	13.0	37.1	69.2	24.3	35.1	41.5	5.8	3.26	2.34	0.08	0.04	0.01	0.08	421	7.5	Vaccine	M
203	5.27	12.2	36.3	68.8	23.1	33.6	32.3	6.73	3.11	3.35	0.15	0.04	0.02	0.07	311	9.3	Vaccine	M
211	4.97	11.7	35.4	71.2	23.6	33.1	45.2	5.38	3.53	1.59	0.09	0.1	0.01	0.06	291	8.2	Vaccine	F
212	5.99	13.4	40.4	67.5	22.3	33.1	58.4	5.76	3.23	2.15	0.20	0.09	0.02	0.08	434	8.0	Vaccine	F
213	5.16	12.2	37.1	71.9	23.5	32.8	77.2	7.64	2.71	4.59	0.14	0.14	0.01	0.05	216	10.5	Vaccine	F

Table S2: Hematology. Hematology data of individual NHPs measured in serum samples collected prior to vaccination on days -21 and 0 (D-21 and D0) and collected on days 1 (D1), 21 (D21), 42 (D42) and 63 (D63) relative to first vaccination.

Day -21

NHP	Alklaline Phosphatase (U/L)	ALT (U/L)	AST (U/L)	LDH (U/L)	GGT (U/L)	Total Bilirubin (mg/dL	Direct Bilirubin (mg/dL)	Total Protein (g/dL)	Glucose (mg/dL)	Albumin (g/dl)	Globulin (g/dL)	A/G Ratio	Blood Urea Nitrogen (mg/dL)	Creatinine (mg/dL)	Creatine Kinase (U/L)	TRIG (mg/dL)	Cholesterol (mg/dL)	Calcium (mg/dL)	Phosphorus (mg/dL)	Sodium (mmol/L	Potassium (mmol/L)	Chloride (mmol/L)	CRP (mg/L)	Group	Sex
101	638	53	34	338	112	0.14	0.02	7.6	115	4.8	2.8	1.7	20	0.7	563	80	185	9.7	7.1	146	3.4	103	0.5	Adjuvant	M
111	509	18	33	375	71	0.18	0.03	7.8	80	4.7	3.1	1.5	18	0.6	320	30	121	9.6	7.1	149	3.7	107	0.3	Adjuvant	F
201	389	18	45	319	66	0.14	0.03	7.2	72.0	4.2	3.0	1.4	22	0.6	2394	56	136	9.3	5.1	147	3.4	105	30.6	Vaccine	M
202	377	46	48	467	70	0.14	0.02	7.4	92	4.7	2.7	1.7	18	0.6	3123	40	159	10.0	5.7	150	4.0	110	0.9	Vaccine	M
203	404	29	33	614	79	0.12	0.02	7.4	93	4.7	2.7	1.7	21	0.7	778	89	141	9.7	4.9	150	3.2	111	0.4	Vaccine	M
211	464	30	32	325	66	0.18	0.04	7.8	81	5.1	2.7	1.9	26	0.9	347	53	164	10.5	6.9	153	4.2	108	0.7	Vaccine	F
212	471	27	42	378	88	0.11	0.03	6.4	64	3.9	2.5	1.6	23	0.5	1561	46	129	8.8	7.3	148	3.5	105	2.1	Vaccine	F
213	496	21	27	250	57	0.11	0.02	7.0	70	4.5	2.5	1.8	20	0.7	753	91	118	9.3	5.5	148	3.7	108	0.5	Vaccine	F

Day 0

NHP	Alklaline Phosphatase (U/L)	ALT (U/L)	AST (U/L)	LDH (U/L)	GGT (U/L)	Total Bilirubin (mg/dL	Direct Bilirubin (mg/dL)	Total Protein (g/dL)	Glucose (mg/dL)	Albumin (g/dl)	Globulin (g/dL)	A/G Ratio	Blood Urea Nitrogen (mg/dL)	Creatinine (mg/dL)	Creatine Kinase (U/L)	TRIG (mg/dL)	Cholesterol (mg/dL)	Calcium (mg/dL)	Phosphorus (mg/dL)	Sodium (mmol/L	Potassium (mmol/L)	Chloride (mmol/L)	CRP (mg/L)	Group	Sex
101	638	53	34	338	112	0.14	0.02	7.6	115	4.8	2.8	1.7	20	0.7	563	80	185	9.7	7.1	146	3.4	103	0.5	Adjuvant	M
111	509	18	33	375	71	0.18	0.03	7.8	80	4.7	3.1	1.5	18	0.6	320	30	121	9.6	7.1	149	3.7	107	0.3	Adjuvant	F
201	389	18	45	319	66	0.14	0.03	7.2	72.0	4.2	3.0	1.4	22	0.6	2394	56	136	9.3	5.1	147	3.4	105	30.6	Vaccine	M
202	377	46	48	467	70	0.14	0.02	7.4	92	4.7	2.7	1.7	18	0.6	3123	40	159	10.0	5.7	150	4.0	110	0.9	Vaccine	M
203	404	29	33	614	79	0.12	0.02	7.4	93	4.7	2.7	1.7	21	0.7	778	89	141	9.7	4.9	150	3.2	111	0.4	Vaccine	M
211	464	30	32	325	66	0.18	0.04	7.8	81	5.1	2.7	1.9	26	0.9	347	53	164	10.5	6.9	153	4.2	108	0.7	Vaccine	F
212	471	27	42	378	88	0.11	0.03	6.4	64	3.9	2.5	1.6	23	0.5	1561	46	129	8.8	7.3	148	3.5	105	2.1	Vaccine	F
213	496	21	27	250	57	0.11	0.02	7.0	70	4.5	2.5	1.8	20	0.7	753	91	118	9.3	5.5	148	3.7	108	0.5	Vaccine	F

Day 21

NHP	Alklaline Phosphatase (U/L)	ALT (U/L)	AST (U/L)	LDH (U/L)	GGT (U/L)	Total Bilirubin (mg/dL	Direct Bilirubin (mg/dL)	Total Protein (g/dL)	Glucose (mg/dL)	Albumin (g/dl)	Globulin (g/dL)	A/G Ratio	Blood Urea Nitrogen (mg/dL)	Creatinine (mg/dL)	Creatine Kinase (U/L)	TRIG (mg/dL)	Cholesterol (mg/dL)	Calcium (mg/dL)	Phosphorus (mg/dL)	Sodium (mmol/L	Potassium (mmol/L)	Chloride (mmol/L)	CRP (mg/L)	Group	Sex
101	611	46	38	402	98	0.15	0.04	7.7	96	5.2	2.5	2.1	21	0.6	335	95	198	9.8	6.7	143	3.3	101	0.8	Adjuvant	M
111	515	17	35	453	78	0.19	0.06	8.0	86	4.9	3.1	1.6	18	0.6	244	46	123	9.8	6.3	145	3.2	103	0.7	Adjuvant	F
201	566	17	33	437	94	0.19	0.06	7.3	66	4.4	2.9	1.5	26	0.5	527	77	140	9.3	5.7	143	3	102	0.4	Vaccine	M
202	392	34	32	519	65	0.17	0.03	7.5	84	4.7	2.8	1.7	22	0.6	443	42	150	9.8	5.2	146	3.5	108	0.0	Vaccine	M
203	384	30	34	663	80	0.11	0.03	7.7	100	4.8	2.9	1.7	22	0.7	291	95	149	9.8	5.3	147	3.2	107	2.0	Vaccine	M
211	349	26	29	306	67	0.14	0.04	7.8	89	5	2.8	1.8	24	0.7	259	69	164	10.0	6.0	148	3.1	107	2.4	Vaccine	F
212	678	22	36	392	112	0.23	0.06	6.9	75	4.5	2.4	1.9	19	0.6	1055	57	126	9.4	6.9	147	3.6	104	2.0	Vaccine	F
213	506	29	27	244	80	0.15	0.04	7.3	47	4.8	2.5	1.9	19	0.7	466	95	148	9.6	5.6	148	3.4	107	0.0	Vaccine	F

Day 42

NHP	Alklaline Phosphatase (U/L)	ALT (U/L)	AST (U/L)	LDH (U/L)	GGT (U/L)	Total Bilirubin (mg/dL	Direct Bilirubin (mg/dL)	Total Protein (g/dL)	Glucose (mg/dL)	Albumin (g/dl)	Globulin (g/dL)	A/G Ratio	Blood Urea Nitrogen (mg/dL)	Creatinine (mg/dL)	Creatine Kinase (U/L)	TRIG (mg/dL)	Cholesterol (mg/dL)	Calcium (mg/dL)	Phosphorus (mg/dL)	Sodium (mmol/L	Potassium (mmol/L)	Chloride (mmol/L)	CRP (mg/L)	Group	Sex
101	603	45	36	337	98	0.17	0.05	7.6	107	5.3	2.3	2.3	19	0.7	877	88	189	9.8	5.7	147	3.3	105	0.0	Adjuvant	M
111	513	19	32	375	71	0.19	0.06	8.0	101	5.0	3.0	1.7	20.0	0.7	239	59	127	9.8	6.5	148	3.3	103	0.0	Adjuvant	F
201	591	21	37	332	99	0.29	0.08	6.7	65	4.5	2.2	2	24	0.6	1059	56	128	9.2	5.6	146	3.4	105	0.0	Vaccine	M
202	406	46	32	345	68	0.13	0.04	7.2	100	4.6	2.6	1.8	22	0.7	338	48	141	9.7	4.7	147	3.5	108	0.0	Vaccine	M
203	450	27	31	531	85	0.12	0.04	7.6	101	4.7	2.9	1.6	19	0.7	353	111	143	650	4.6	147	3.3	107	0.0	Vaccine	M
211	368	28	25	245	73	0.09	0.04	6.4	70	4.1	2.3	1.8	24	0.6	406	60	136	9.4	6.2	149	3.2	110	0.0	Vaccine	F
212	691	26	34	263	116	0.21	0.06	6.8	85	4.3	2.5	1.7	21	0.7	825	71	116	9.7	6.6	149	3.4	104	0.0	Vaccine	F
213	590	29	24	190	85	0.17	0.05	7.0	52	4.8	2.2	2.2	20	0.7	249	104	140	9.7	4.4	148	3.4	108	0.0	Vaccine	F

Day 63

NHP	Alklaline Phosphatase (U/L)	ALT (U/L)	AST (U/L)	LDH (U/L)	GGT (U/L)	Total Bilirubin (mg/dL	Direct Bilirubin (mg/dL)	Total Protein (g/dL)	Glucose (mg/dL)	Albumin (g/dl)	Globulin (g/dL)	A/G Ratio	Blood Urea Nitrogen (mg/dL)	Creatinine (mg/dL)	Creatine Kinase (U/L)	TRIG (mg/dL)	Cholesterol (mg/dL)	Calcium (mg/dL)	Phosphorus (mg/dL)	Sodium (mmol/L	Potassium (mmol/L)	Chloride (mmol/L)	CRP (mg/L)	Group	Sex
101	650	38	33	324	102	0.22	0.06	6.9	6.8	4.7	2.2	2.1	17	0.5	405	69	171	9.5	6.3	148	3.5	106	1.9	Adjuvant	M
111	468	22	43	472	69	0.27	0.07	7.1	77	4.3	2.8	1.5	21	0.5	841	52	111	9.4	6.3	147	3.2	103	2.3	Adjuvant	F
201	595	17	33	337	94	0.26	0.07	6.5	79	4.2	2.3	1.8	24	0.6	465	46	121	9.2	6.6	147	3.2	105	2.6	Vaccine	M
202	405	38	34	385	74	0.18	0.05	7.1	90	4.7	2.4	2.0	21	0.7	2376	61	150	9.8	4.9	149	3.3	107	1.6	Vaccine	M
203	480	25	29	477	91	0.24	0.05	7.7	74	4.6	3.1	1.5	19	0.6	301	98	151	9.8	4.8	151	3.3	108	1.8	Vaccine	M
211	378	32	28	266	76	0.15	0.05	6.9	83	4.5	2.4	1.9	21	0.5	198	44	143	9.7	5.8	151	3.2	109	1.5	Vaccine	F
212	736	25	35	313	118	0.33	0.09	6.8	82	4.2	2.6	1.6	20	0.6	1321	42	114	9.6	6.8	149	3.5	105	3.9	Vaccine	F
213	555	28	32	274	88	0.23	0.06	6.6	49	4.3	2.3	1.9	19	0.6	1737	63	131	9.3	5.4	149	3.3	109	1.6	Vaccine	F

Table S3: Serum Chemistry. Serum chemistry data of individual NHPs measured in serum samples collected prior to vaccination on days -21 and 0 (D-21 and D0) and collected on days 1 (D1), 21 (D21), 42 (D42) and 63 (D63) relative to first vaccination.

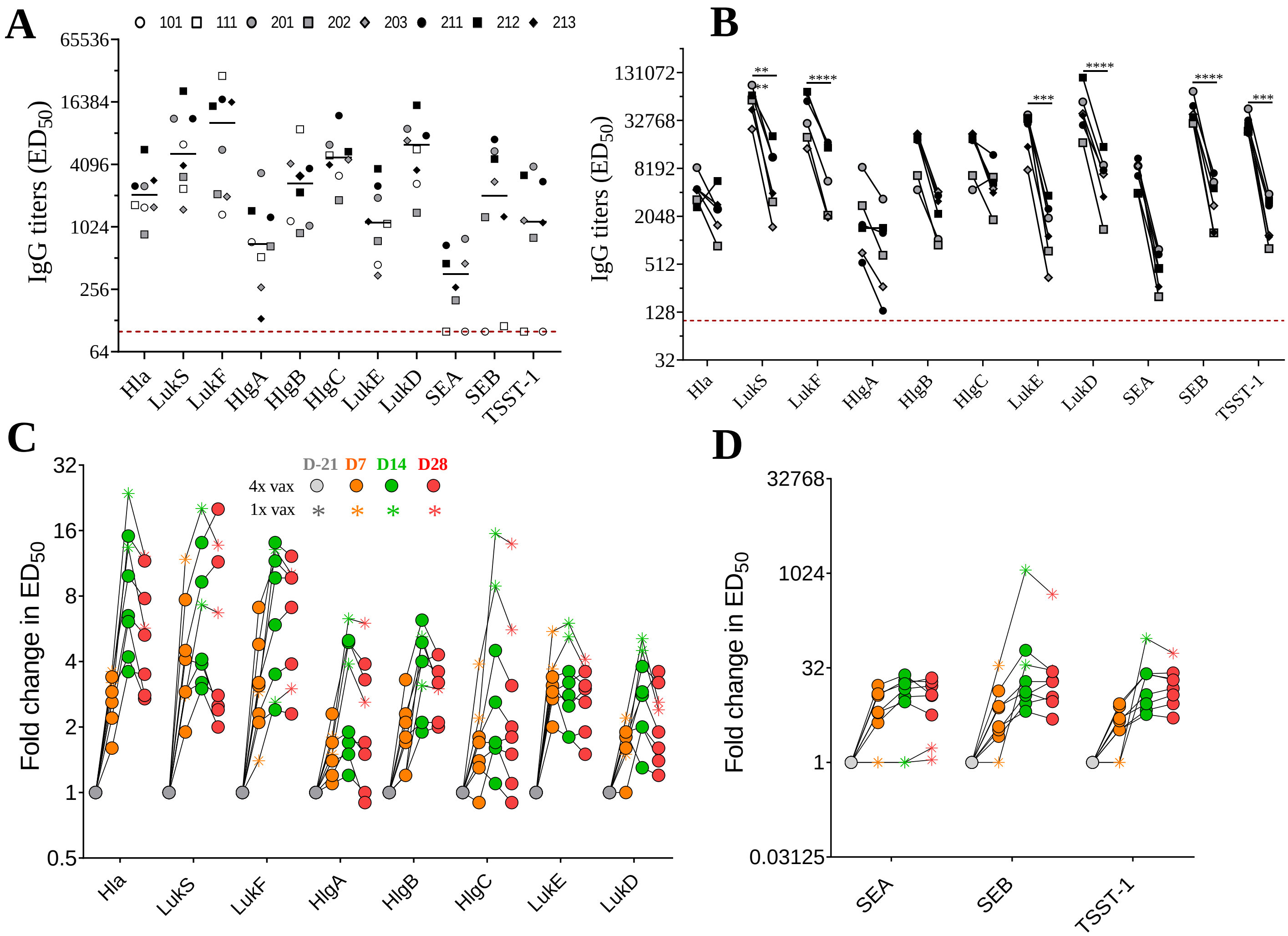

Figure S1: Plasma IgG titers after third booster. (A) Pre-existing IgG titers of NHPs 21 days prior to the third booster, represented as plasma dilution that elicited 50% binding titers (ED_{50}) (B) Comparison of Day 63 and Day 168 IgG titers to depict the drop in titers over the 100 days gap period between the second and third booster (C) Fold increase of IgG titers of NHP sera collected on days -21 (day 168), 7 (day 196), 14 (day 203) and 28 (day 217) towards wildtype pore-forming toxin Hla, PVL subunits LukS and LukF, HlgAB/CB subunits HlgA, -B, and –C, and LukED subunits LukE and LukD and (D) towards wildtype superantigen toxins SEA, SEB and TSST-1. Colored symbols reflect indicated days post vaccination. Plasma of 4x immunized NHPs depicted as circled symbols and plasma of 1x immunized NHPs depicted as star symbols.

A

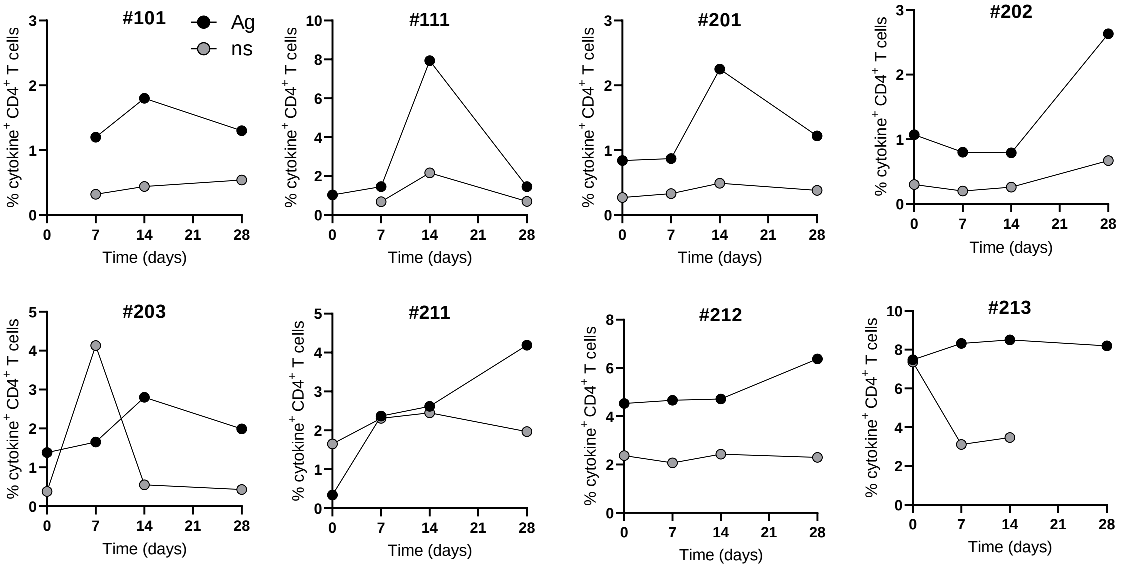

Figure S2: Kinetics of cytokine+ CD4 T cells. PBMCs of 1x vaccinated (#101 and #111) and 4x vaccinated NHPs (#201, 202, 203 and #211, 212, 213) were stimulated *ex vivo* with 4-component vaccine and frequency of cytokine positive CD4 T cells was determined by flow cytometry.
